# Hydrogen and nitrogen codoping of anatase TiO_2_ for efficiency enhancement in organic solar cells

**DOI:** 10.1038/s41598-017-18051-0

**Published:** 2017-12-19

**Authors:** Maria Vasilopoulou, Nikolaos Kelaidis, Ermioni Polydorou, Anastasia Soultati, Dimitris Davazoglou, Panagiotis Argitis, Giorgos Papadimitropoulos, Dimitris Tsikritzis, Stella Kennou, Florian Auras, Dimitra G. Georgiadou, Stavros-Richard G. Christopoulos, Alexander Chroneos

**Affiliations:** 1 National Center for Scientific Research Demokritos, Institute of Nanoscience and Nanotechnology (INN), 15310 Agia Paraskevi Athens, Greece; 2 Faculty of Engineering, Environment and Computing, Coventry University, Priory Street, Coventry, CV1 5FB United Kingdom; 3 Department of Physics, University of Patras, 26504 Patras, Greece; 4 Department of Chemical Engineering, University of Patras, 26504 Patras, Greece; 5 Cavendish Laboratory, University of Cambridge, Cambridge, CB3 0HE United Kingdom; 6 Department of Physics & Centre for Plastic Electronics, Imperial College, London, SW7 2AZ United Kingdom; 7 Department of Materials, Imperial College, London, SW7 2AZ United Kingdom

## Abstract

TiO_2_ has high chemical stability, strong catalytic activity and is an electron transport material in organic solar cells. However, the presence of trap states near the band edges of TiO_2_ arising from defects at grain boundaries significantly affects the efficiency of organic solar cells. To become an efficient electron transport material for organic photovoltaics and related devices, such as perovskite solar cells and photocatalytic devices, it is important to tailor its band edges via doping. Nitrogen p-type doping has attracted considerable attention in enhancing the photocatalytic efficiency of TiO_2_ under visible light irradiation while hydrogen n-type doping increases its electron conductivity. DFT calculations in TiO_2_ provide evidence that nitrogen and hydrogen can be incorporated in interstitial sites and possibly form N_i_H_i_, N_i_H_O_ and N_Ti_H_i_ defects. The experimental results indicate that N_i_H_i_ defects are most likely formed and these defects do not introduce deep level states. Furthermore, we show that the efficiency of P3HT:IC_60_BA-based organic photovoltaic devices is enhanced when using hydrogen-doping and nitrogen/hydrogen codoping of TiO_2_, both boosting the material n-type conductivity, with maximum power conversion efficiency reaching values of 6.51% and 6.58%, respectively, which are much higher than those of the cells with the as-deposited (4.87%) and nitrogen-doped TiO_2_ (4.46%).

## Introduction

Metal oxides such as titanium dioxide (TiO_2_) have been intensively investigated for more than four decades because of their strong catalytic activity, high chemical stability and long lifetime of photon generated carriers^1–10^. Anatase exhibits the highest photocatalytic activity of the polymorphs of TiO_2_, however, it is constrained to the limited ultraviolet range (UV irradiation is only 5%) of the solar spectrum due to its large band gap (3.2eV)^7^. For a photocatalyst to achieve high efficiency, its band gap should be around 2.0 eV, whereas the position of the band edges should be consistent with the redox potential of water^11^. A way to reduce the band gap is doping, with nitrogen (N) atom being a particularly promising p-type dopant^3,12^.

Hydrogen (H) is a small atom so it can diffuse easily in inorganic compounds occupying interstitial sites. It does not induce significant structural expansion, can modify the band gap, enhance the photocatalytic activity^13^, induce insulator-to-conductor transitions^14^, provide free electrons^15^, and interact with intrinsic defects such as oxygen vacancies^16^. H can be introduced in TiO_2_ during synthesis or by immersion in water^12^. Interestingly, previous theoretical studies have shown that hydrogen can substitute for oxygen (termed as substitutional H, H_O_) leading to *n*-type conductivity^17,18^. H doping has recently been established as an effective strategy for improving the capacitive properties of TiO_2_ for application in supercapacitors^19^. Additionally, the emergence of a highly H doped TiO_2_ (black titania) nanomaterial has triggered world-wide research interest, because of its substantially enhanced solar absorption and improved photocatalytic activities^20^. Notably, an effective way to achieve H doping of TiO_2_ is by subjecting it to post-annealing in a reducing environment i.e. forming gas, a 90:10 per volume mixture of nitrogen:hydrogen, which, however, indicates that simultaneous N and H codoping is possible in this case.

On the other hand, organic solar cells (OSCs) have attracted growing attention over the past decades because of their advantages in fabricating renewable, low cost, lightweight, flexible and large size light harvesting solar cells^21,22^. TiO_2_ has been recognized as a promising electron transport layer (ETL) used to enhance the electron extraction efficiency in OSCs due to its adequate electron mobility, excellent optical transparency, and solution-based fabrication^23–25^. However, there are limitations in increasing OSCs efficiency by using as-deposited TiO_2_ resulting from its low n-type conductivity which hampers efficient electron transport towards cathode contact and from high recombination rates of photogenerated electron-hole pairs through trap states present at the metal oxide surface^26,27^. To overcome these limitations and produce efficient metal oxide electron transport layer modification of TiO_2_ has been developed through UV irradiation^28,29^ which, however, may have a degradation effect thus hindering the device lifetime^30^. It would, therefore, be highly desirable to develop appropriate n-type doped TiO_2_-based electron transport materials to boost the efficiency of OSCs and of related devices.

Recently, our group demonstrated the beneficial effect of H doping of zinc oxide (ZnO), which represents one of the commonly used electron transport materials in OSC technology^31,32^. However, the possible effectiveness of H as well as of N doping of TiO_2_ used as ETLs in OSCs has not been demonstrated thus far. Moreover, the investigation of possible interaction of H and N dopants to reveal if simultaneous codoping of TiO_2_ is possible is of high priority since the common reducing environment of TiO_2_ contains both potential dopants. Here, we explore, **from both theoretical and experimental points of view**, the influence of H and N doping as well as of N, H codoping of anatase TiO_2_ for application in OSCs. We show that while N causes p-type doping since it creates deep-lying intergap states that act as recombination centers which are detrimental for the device performance, H doping and, especially, N, H codoping cause n-type doping and, therefore, can be effective ways to increase the electron conductivity and passivate surface dangling bonds of TiO_2_ leading to high-efficiency organic photovoltaics. In particular, inverted OSC devices based on photoactive blends of poly(3-hexylthiophene) (P3HT) with indene-C_60_ bisadduct (IC_60_BA) substantially enhanced their photovoltaic performance reaching power conversion efficiency (PCE) values of 6.51% and 6.58%, respectively, when using the H-doped and N, H-codoped TiO_2_ ETLs. This represents an increase in performance by 34% and 35%, respectively, over the reference cells comprising an unmodified TiO_2_ layer (4.87%). On the contrary, N doping of TiO_2_ resulted in a decreased efficiency of 4.46% which represents an 8% efficiency drop as compared to the reference cell.

## Results and Discussion

### Theoretical results and discussion

TiO_2_ has three polymorphs namely rutile, anatase and brookite. Both rutile and anatase are tetragonal with space groups P4_2_/mnm and I4/amd respectively^33,34^. The lattice parameters of anatase TiO_2_ are calculated at a = 3.806 Å and c = 9.724 Å in excellent agreement with the experimental neutron diffraction results (a = 3.782 Å and c = 9.502 Å)^33^ and theoretical results (a = 3.729–3.801 Å and c = 9.480–9.818 Å)^34^.

**Figure 1:**
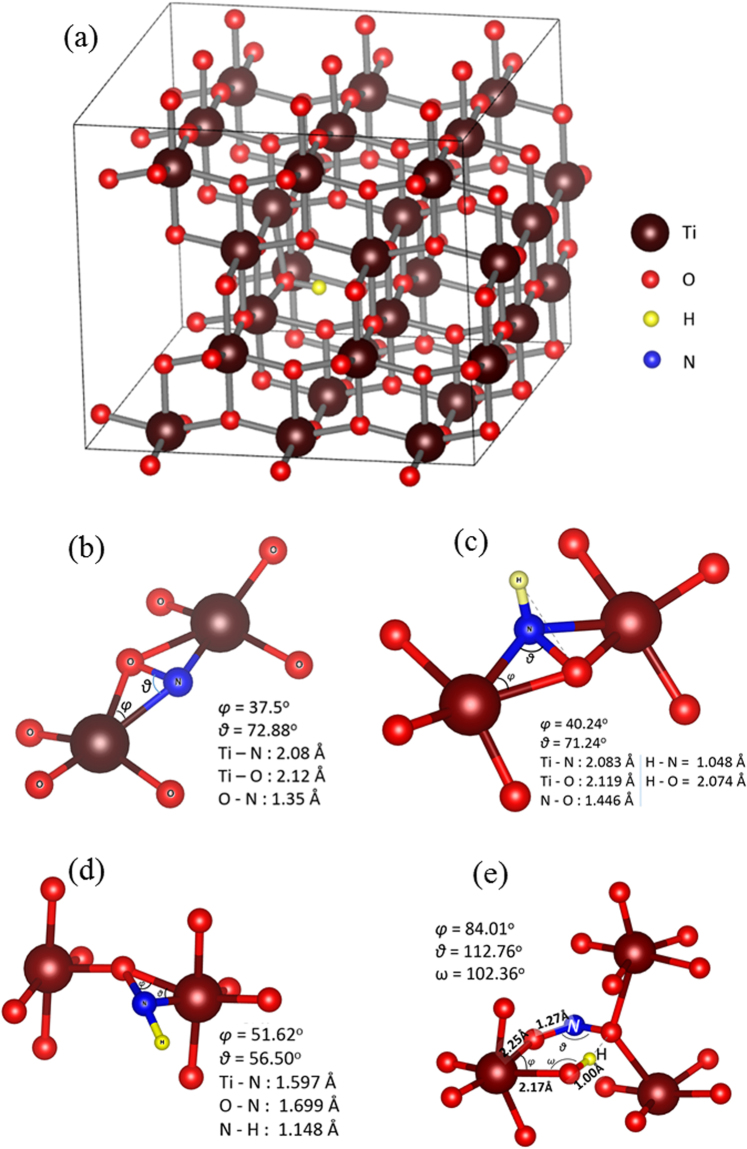
The structure of minimum energy defects in anatase. (**a**) H_i_ in the supercell, (**b**) the N_i_, (**c**) N_i_H_i_ (**d**) N_i_H_O_ and (**e**) N_Ti_H_i_.

Although anatase and rutile have the same chemical composition, they differ in the coordination environments (i.e. chemical bonding) and this leads to different ionization potentials and chemical affinities^35^. Interestingly, in a recent report Luttrell *et al* ^36^. studied epitaxial TiO_2_ films to consider why anatase is a better photocatalyst as compared to rutile. This study determined that charge carriers that were excited deeper in the bulk contributed more to surface reaction in anatase as compared to rutile^36^. Considering this, the present study will focus on H and N defects in bulk anatase discussing wherever appropriate, recent DFT results concerning the anatase TiO_2_ surface^37^. It is calculated using DFT that atomic hydrogen can be a substitutional defect occupying oxygen sites (referred to as H_O_). This is in agreement with the present experimental results which identified only Ti^4+^ states (as discussed in the section referred to the synthesis of TiO_2_ materials) and previous DFT studies^38^. Additionally, we considered interstitial hydrogen (H_i_) and developed an algorithm to track the minimum energy interstitial sites using an extensive search. We calculate that H_i_ resides 0.99 Å from the nearest oxygen atom (Fig. 1(a)). This is in agreement with previous DFT results^39^.

**Figure 2:**
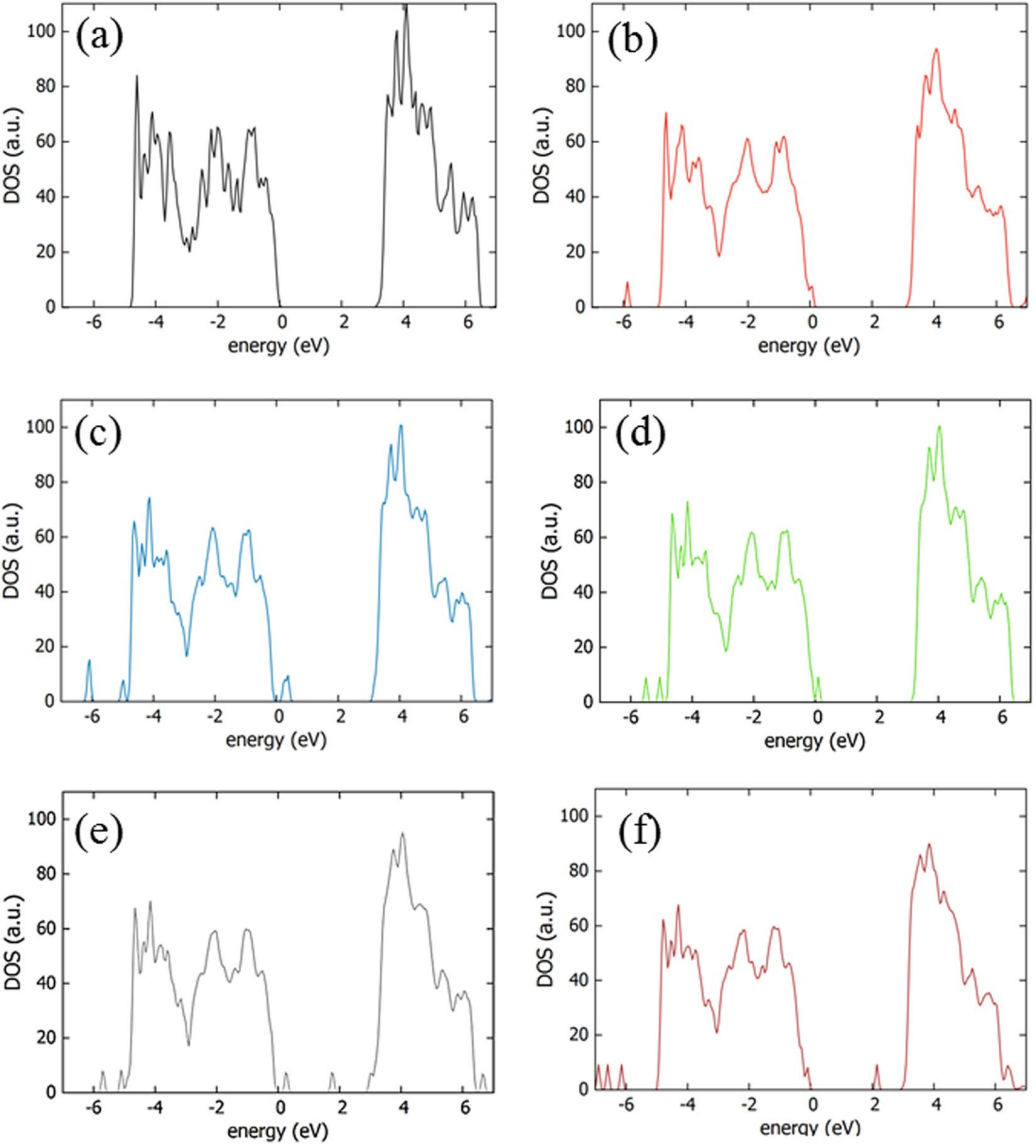
DOS of (**a**) undoped supercell, (**b**) H_i_, **(c)** the N_i_, (**d**) N_i_H_i_ (**e**) N_i_H_O_ and (**f**) N_Ti_H_i_ defects of TiO_2_.

Next, the electronic density of states (DOS) for anatase TiO_2_ was calculated in order ito understand the electronic structure and nature of the band edge wavefunctions (Fig. 2(a)). The valence band edge of the material is dominated by O 2p, and the conduction band edge is formed from Ti 3d orbitals. The band gap is calculated to be 3.1 eV in agreement with the experimentally measured one (3.2 eV). Adding H_i_ atoms to form multi-hydrogen clusters is energetically unfavourable (for example in the H_i_H_i_ pair the atoms repel each other with 1.14 eV). DOS reveal that the introduction of H leads only to a small band gap narrowing (refer to Fig. 2(b)) consistently with the experimental results shown below.

The introduction of nitrogen was considered at both a Ti site (N_Ti_) or as an interstitial (N_i_, refer to Fig. 1(b)). For N_i_ a density of new states (refer to Fig. 2(c)) is observed above the valence band maximum, VBM (i.e. p-type doping); this is attributed to the N 2p states lying just above the O 2p states.

In codoped anatase it should be expected that N and H defects can interact. Here we have performed an extensive investigation of the different possibilities that the interstitial and/or substitutional hydrogen and nitrogen defects can associate. Figure 1(c–e) represents the N_i_H_i_, N_i_H_O_ and N_Ti_H_i_ defect pairs. The N_i_H_i_ defect pair is bound (−0.33 eV) when the two interstitials are close at N-H = 1.05 Å (Fig. 1(c)) as compared to when the N-H distance is 8.07 Å. The N_i_H_O_ is calculated to have a binding energy of −3.77 eV (energy difference between configurations being at N-H = 0.93 Å and N-H = 7.96 Å), however, its concentration will be limited by the number of available oxygen vacancies (as there as required for H_O_ to form). Therefore, although the majority of oxygen vacancies should be occupied by atomic hydrogen^18,32^, the concentration of N_i_H_O_ will depend upon the growth conditions. Finally, the N_Ti_H_i_ defect is bound with −1.45 eV (energy difference between configurations being at N-H = 2.88 Å and N-H = 10.87 Å). In essence, N_Ti_ defect will capture migrating H_i_, but again this defect will require Ti vacancies to form in the first place.

The DOS reveal that the N_i_H_O_ and N_Ti_H_i_ defects (Fig. 2(e–f)) introduce levels at about 1.05 eV and 0.75 eV below the conduction band minimum, CBM. More importantly, N_i_H_O_ defect introduces a pronounced density of states above the VBM resulting in undesirable p-type doping. These deep levels, in essence, may act as recombination centers of photo-excited electron-hole pairs and consequently can lower the efficiency of OSCs using TiO_2_ ETLs. Therefore, H,N codoped TiO_2_ should be grown under O-rich conditions (to provide a stoichiometric TiO_2_ as the starting material) to avoid the formation of oxygen vacancies and thus N_i_H_O_ defects.

### Synthesis and characterization of H and N doped and codoped TiO_2_ materials

Solution-processed anatase TiO_2_ films were converted into H-doped (referred to as TiO_2_:H), N-doped (referred to as TiO_2_:N), or codoped (referred to as TiO_2_:H,N) TiO_2_ films by annealing at 550 °C in N_2_, H_2_, or in forming gas, respectively. In Fourier transform infra-red (FTIR) transmittance spectra (refer to Fig. S1, Supporting Information) taken in the above films exhibiting the same thickness of ~40 nm (refer to Fig. S2), apart from a broad band at the lower wavenumbers which is due to Ti-O bonds, the spectral features due to defect species are negligible; however, hydrogen and forming gas annealing seems to increase the intensity of the broad peak at 3000–3600 cm^−1^ in the region were stretching of hydroxyl group ν(OH) appears which is an indication for the insertion of hydrogen within the oxide lattice (i.e., H doping). This peak is decreased after annealing in nitrogen. From the analysis of FTIR spectra the incorporation of hydrogen dopants within the material after annealing in hydrogen and forming gas can be concluded; however, the content of H dopants cannot be estimated from those spectra since the contribution of water in the intensity of the peak around 3500 cm^−1^ cannot be excluded. Notably, the incorporation of hydrogen within the material causes some alteration in its crystallinity, especially in the hydrogen annealed sample (refer to Fig. S3). Moreover, TiO_2_ samples exhibit very small band gap narrowing with H doping (from 3.20 to 3.14 eV) while the band gap narrowing is more pronounced with N doping (3.10 eV) (Fig. S4). N and H codoping only has a minor effect on the band gap value (3.13 eV). These results are in agreement with those obtained by Pan *et al* ^12^.

X-ray photoelectron spectroscopy (XPS) analysis of the surface elementary composition of the formulated materials confirms that N was detected in both samples deposited in environment containing N (Fig. S5). This observation indicates that N doping takes place after annealing in nitrogen and forming gas. Only Ti^4+^ states were identified in the starting material (Fig. S6), revealing that the as-deposited TiO_2_ film is highly stoichiometric^40^. In addition, Ti^4+^ states were also detected in the annealed samples (Fig. S6). Since we do not find localized Ti^3+^ states in our annealed samples, it can be concluded that on the sample surface there are no other Ti states. Figure S7 represents the O*1s* spectra of TiO_2_ samples. The peak at binding energy 530.4 ± 0.1 eV (fittings are not included here) corresponds to O-Ti bonds in TiO_2_. A second component is always present which is broader at binding energy 531.6 ± 0.1 eV due to Ti-OH bonds. The broadening in the O 1 s peaks of nitrogen containing samples could be due to the combination of hydroxyl groups and N-O-Ti bonds present in these samples.

**Figure 3:**
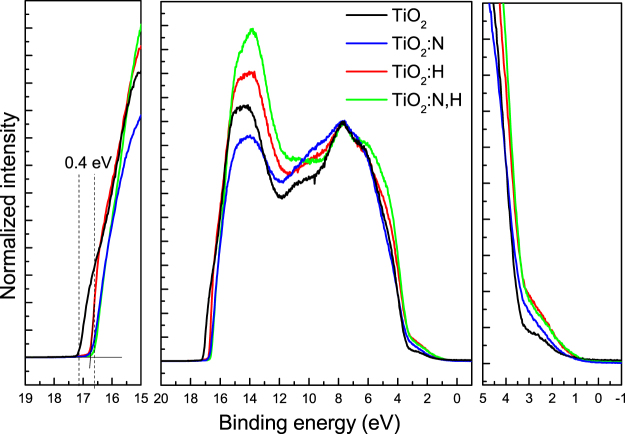
UPS measurements of different TiO_2_ samples.

The surface electronic structure of different TiO_2_ materials was evaluated by ultra-violet photoelectron spectroscopy (UPS) measurements shown in Fig. 3, which reveal that the valence band of TiO_2_ samples remain almost unaffected.

The valence band of the TiO_2_ sample consists of a broad peak at about 6 eV and a narrow peak at about 8 eV, which correspond to π (non-bonding) and σ (bonding) Ti3d-O 2p orbitals^41^. An additional small peak, located at 10.6 eV, is observed in all spectra and it is more pronounced in the hydrogenated samples. This feature suggests the presence of an adsorbate such as -OH on the surface and it is correlated with the 3σ of such molecules. However, this feature is more pronounced in the spectra of samples annealed either in hydrogen or in forming gas, an indication of H doping in these cases. The VBM of TiO_2_ is located at ~3.2 eV below the Fermi level while its work function was found equal to 4.1 eV. As its band gap is estimated to be 3.2 eV the bottom of its conduction band locates at ~4.1 eV indicating the n-type character of the TiO_2_ film. The VBM of the doped TiO_2_ films slightly shifts to 3.0 eV before the Fermi level while for the TiO_2_:H and TiO_2_:N,H samples there is an increase in the W_F_ value by 0.3 eV which is due to the adsorbed -OH species on the surface. The work function of N containing samples is increased by 0.4 eV in comparison with the TiO_2_ sample due to the p-type doping character of N. The results provide evidence that treatment of TiO_2_ with hydrogen and, especially, with nitrogen results in the creation of gap states above the VBM and, concomitantly, in a decrease of the effective band gap of the semiconductor^42^.

**Figure 4:**
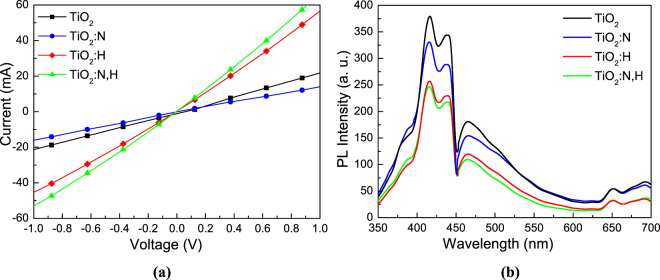
(**a**) Current-voltage characteristics of diodes with the structure FTO/TiO_2_/Al for 40 nm thick TiO_2_ layers treated under different environment. (**b**) PL spectra of TiO_2_ films deposited on FTO substrates with and without N and H doping and N,H codoping.

H intercalation within a metal oxide such as TiO_2_ is expected to increase its n-type conductivity as well as to passivate surface defects^43,44^. To support this argument we next measured the current-voltage characteristics of devices with the structure glass/FTO/TiO_2_/Al (Fig. 4(a)). The thickness of TiO_2_ was measured at 40 nm in all cases. The significant increase in the slopes of I-V curves upon H doping and N,H codoping of TiO_2_ witnesses the improved n-type conductivity of the latter. On the contrary, reduction of n-type conductivity is concluded from the reduced slope of the I-V curve of the TiO_2_:N embedding diode.

The photoluminescence (PL) emission spectrum of TiO_2_ results from the recombination of free carriers; therefore, these spectra are used to understand the effect of surface traps on photo-generated electrons and holes in TiO_2_ samples^45^. The PL spectra of as-deposited, N and H doped and codoped TiO_2_ samples in the wavelength range of 350–700 nm with the excitation at 325 nm are shown in Fig. 4(b). The shape of the emission spectra is very similar consisting of two broad peaks located at about 400–450 nm and at 450–600 nm, respectively. These two peaks result from surface defects of the TiO_2_ samples. The PL peaks intensity of the H doped and N,H codoped TiO_2_ decreases as compared to the as-deposited TiO_2_ which is an indication of lower recombination rate of electrons and holes due to effective passivation of the surface (and bulk) defects of these samples upon H doping. Note that our doped TiO_2_ samples retained their electrical and optical characteristics presented above when stored in air for a long period of time (data not shown).

### Application of H and N doped and codoped TiO_2_ in organic solar cells

**Figure 5:**
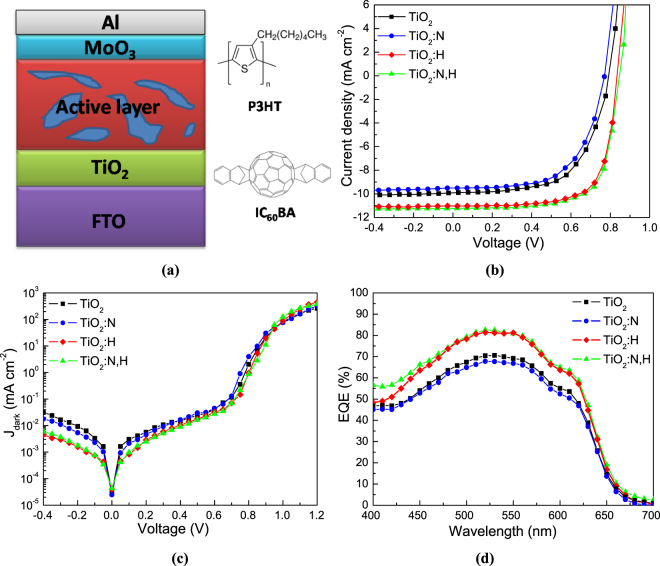
(**a**) The inverted organic solar cell architecture and the chemical structures of organic semiconductors used in this study. **(b)** Current density versus voltage (J-V) characteristics of P3HT:IC_60_BA-based devices using as-deposited TiO_2_ and doped TiO_2_ films upon 1.5 AM illumination. (**c**) Dark J-V curves and (d) EQE measurements taken on devices embedding different TiO_2_ ETLs.

The configuration of the inverted polymer solar cells using TiO_2_ electron transport layers is illustrated in Fig. 5(a), where the chemical structures of organic semiconductors (P3HT and IC_60_BA) used in this study are also shown. Figures 5(b),(c) represent the J-V characteristics under full 1.5 AM simulated solar illumination and in dark, respectively, of devices using photoactive blends based on P3HT:IC_60_BA with as-deposited, N and H doped and codoped TiO_2_ ETLs, respectively. The corresponding electrical output parameters of those devices are summarized in Table 1. The external quantum efficiency (EQE) characteristics of the same devices are presented in Fig. 5(d). For the reference P3HT:IC_60_BA-based device with the as-deposited TiO_2_ layer a PCE of 4.87% is obtained while N p-type doping of TiO_2_ results in a PCE of 4.46%, which is 8% lower as compared to the reference cell. On the contrary, H doping and N and H codoping result in PCEs up to 6.51% and 6.58%, respectively, representing a 34% and 35% improvement as compared to the control device. From the photovoltaic parameters presented in Table 1 becomes evident that by using TiO_2_:H and, especially, TiO_2_:N,H ETLs a simultaneous improvement in the open-circuit voltage (V_oc_), short-circuit current density (J_sc_) and fill factor (FF) of the devices is obtained. In addition, the dark J-V characteristics (shown here in semi-logarithmic scale, Fig. 5(c)) are improved in the case of TiO_2_:H and TiO_2_:N,H, compared to the reference and, especially, to TiO_2_:N-based one. Importantly, the reverse saturation current density decreases significantly in the case of hydrogen containing TiO_2_-based devices. These results suggest that H doping and N,H codoping of TiO_2_ reduces reverse leakage and shunt current while it also facilitates electron extraction probably due to an increase of the built-in field and/or due to enhanced n-type conductivity of the metal oxide. Indeed, upon O–H bond formation, an electron is donated from the Ti–O(H)–Ti species, which results in n-type doping of the TiO_2_ thus improving its n-type conductivity. Both reduction of the reverse saturation current and increase of the turn-on voltage contribute to the increase of the V_oc_ of the H-doped TiO_2_-based solar cells. In addition, the devices with the TiO_2_:H and TiO_2_:N,H layers exhibit significantly reduced series and larger shunt resistances (refer to Table 1) verifying the overall higher quality of the corresponding diodes and explaining the enhanced J_sc_ and FF obtained in these devices. The large improvement in J_sc_ is further supported by the external quantum efficiency (EQE) measurements (refer to Fig. 5(d)). In addition, the EQE spectra of the devices were integrated from 300 to 800 nm and J_sc(EQE)_ values were calculated from the integration and listed in Table 1. Notably, these values are quite consistent with the J_sc_ obtained from J–V characteristics (especially for the devices with the H doped and N,H codoped TiO_2_) under illumination indicating the reliability of the measured J_sc._

**Table 1:** Performance parameters of organic solar cells with the structure FTO/ETL /P3HT:IC_60_BA/MoO_3_/Al. Mean values and standard deviations were extracted from a batch of 18 identical devices.

ETL	J_sc_(mA cm^−2^)	J_sc(EQE)_(mA cm^−2^)	V_oc_(V)	FF	PCE(%)	R_s_(Ω cm^2^)	R_sh_(Ω cm^2^)
TiO_2_	9.95 ( ± 0.19)	9.28	0.79 ( ± 0.01)	0.62 ( ± 0.01)	4.87 ( ± 0.20)	3.9	1900
ΤιΟ_2_:N	9.50 ( ± 0.15)	8.94	0.77 ( ± 0.01)	0.61 ( ± 0.01)	4.46 ( ± 0.17)	4.7	2100
ΤιΟ_2_:H	11.04 ( ± 0.15)	10.70	0.83 ( ± 0.01)	0.71 ( ± 0.01)	6.51 ( ± 0.16)	2.6	3900
TiO_2_:N,H	11.19 ( ± 0.17)	11.11	0.84 ( ± 0.01)	0.70 ( ± 0.01)	6.58 ( ± 0.18)	2.7	4050

Similar efficiency enhancement was obtained in organic solar cells using blends of P3HT donor with [6,6]-phenyl-C_70_butyric acid methyl ester (PC_70_BM) acceptor (Fig. S8) thus proving the universality of the present approach. From these results, it becomes evident that H doping of TiO_2_, especially in the presence of nitrogen defects (i.e. N,H codoping), is an effective way to significantly improve the operational characteristics and overall performance of OSCs using TiO_2_ ETLs while a simple nitrogen doping may negatively affect the device performance. According to our theoretical predictions and experimental results one should argue that, the presence of interstitial nitrogen (N_i_) within the material may be beneficial for the incorporation of the non-bonded hydrogen atoms via the formation of N_i_H_i_ bonds thus suppressing the p-type character of N doping while simultaneously increasing the amount of H dopants in TiO_2_.

**Figure 6:**
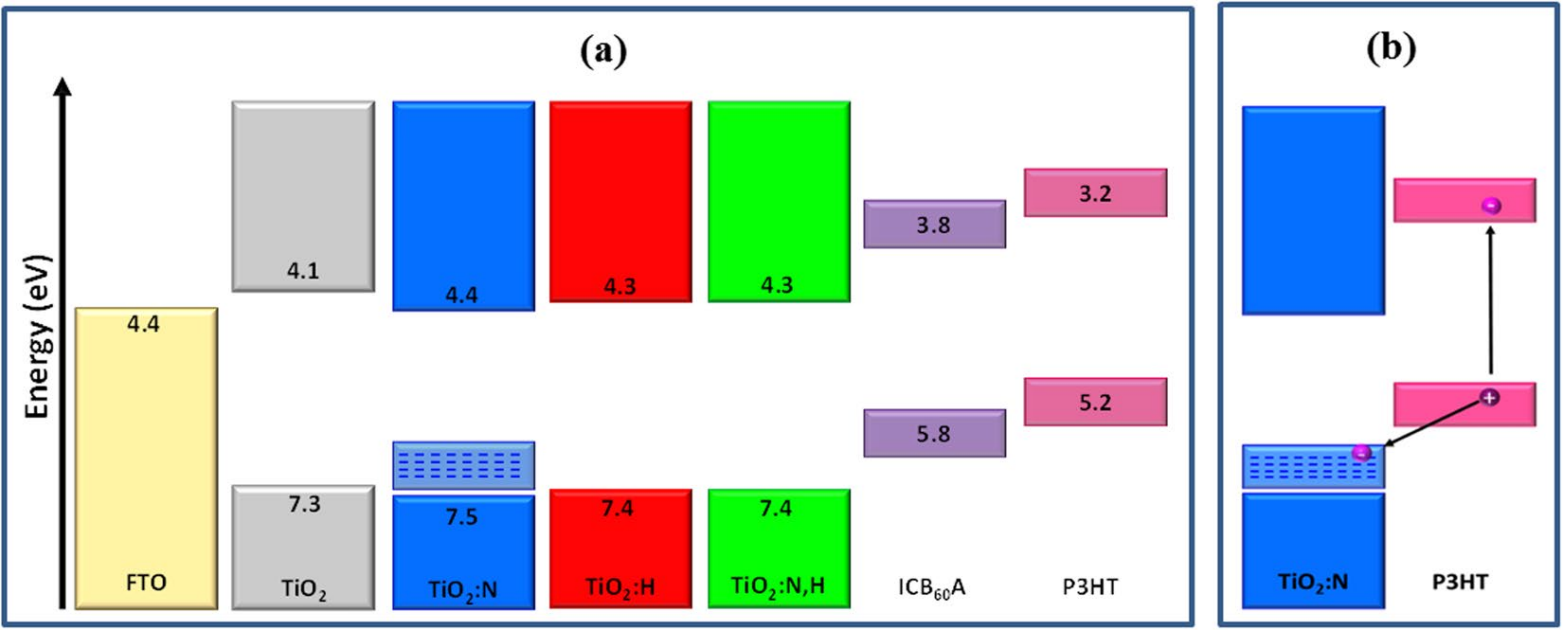
(**a**) Energy level diagram of cathode interfaces with different TiO_2_ ETLs. (**b**) Illustration of possible hole recombination process occurring at the TiO_2_:N/P3HT interface.

To shed more light on the mechanisms responsible for enhanced device performance when using H doped and N,H codoped TiO_2_ ETLs we next constructed the energy level diagram (based on UPS and absorption measurements) of various interfaces before contact (considering vacuum level alignment) at the cathode side of the device. From Fig. 6(a) it becomes evident that in all cases the lowest unoccupied molecular orbital (LUMO) of IC_60_BA acceptor lies above the conduction band minimum of TiO_2_ which indicates that electron extraction occurs via transport of electrons through the conduction band of TiO_2_. In the n-type hydrogen-doped oxides (TiO_2_:H and TiO_2_:N,H) the increase of electron conductivity highly facilitates electron extraction. However, p-type doping of TiO_2_:N sample causes the filling of localized states lying above the VBM as predicted by DFT calculations. Figure 6(b) shows that these localized states are probably aligned with the highest occupied molecular orbital (HOMO) of P3HT. As a result, after photoexcitation of P3HT through light absorption a high hole recombination rate is expected at the TiO_2_:N/P3HT interface which significantly suppresses the device photocurrent. Note that surface states of TiO_2_ arising from oxygen vacancies have also been proven to act as recombination centers thus deteriorating the device current and V_oc_^30^. Passivation of such defect states via hydrogen doping and codoping is expected to be beneficial for electron extraction/collection and overall device performance. Based on the above, one should also conclude that N_i_H_i_ defects are rather formed in the codoped oxide because N_i_H_O_ and N_Ti_H_i_ are predicted to introduce deep level states, exactly as N_i_ does, which are expected to deteriorate the device performance which was not observed in our devices embedding N,H codoped TiO_2_.

**Figure 7:**
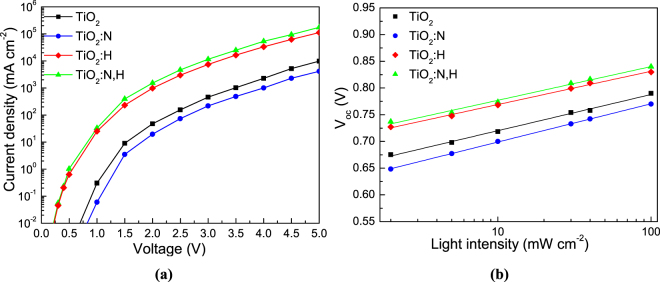
(**a**) Current density-voltage (J−V) curves (measured in dark) in semi-log plot obtained in electron-only devices with the structure: glass/FTO/TiO_2_/P3HT:IC_60_BA/Al where TiO_2_ ETLs are as-deposited N, H or N,H co-doped. (**b**) Dependence of V_oc_ on 1.5 AM illuminated light intensity of P3HT:IC_60_BA-based organic solar cells using different TiO_2_ ETLs.

To further elucidate the effect of N and H doping and codoping of TiO_2_ on the optoelectronic properties of the P3HT:IC_60_BA-based OSCs, we examined the charge transport properties by measuring electron-only devices with the structure: FTO/TiO_2_/P3HT:IC_60_BA/Al. The J-V characteristics are shown in semi-logarithmic plots in Fig. 7(a) and reveal a substantial increase in electron current after the insertion of H dopants in TiO_2_. This improvement in electron current density can be attributed to the significant passivation of surface defect states as well as to increase of n-type conductivity of TiO_2_ upon H doping and is further improved in the case of N,H codoping.

In addition, as shown in Fig. 7(b), where a log-linear plot of V_oc_ as a function of the light intensity is presented, the slope values of V_oc_ were decreased from 1.14 to 1.07 and 1.05 k_B_T/q upon H doping and N,H codoping, where k_B_ is Boltzmann’s constant, T is the absolute temperature, and q is the elementary charge. On the contrary, the slope increases to 1.21 k_B_T/q upon N doping of TiO_2_ ETL. This confirms that the insertion of H dopants into the TiO_2_ lattice significantly reduced trap-assisted recombination at open circuit by passivating surface defects on TiO_2_, leading to an enhanced electron collection (and consequently, J_sc_), FF and PCE values^46^.

**Figure 8:**
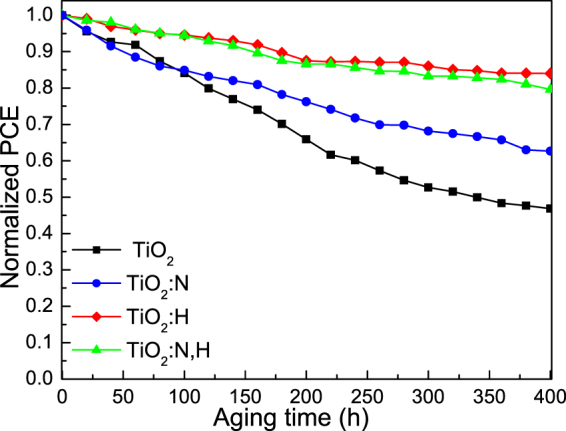
Stability measurements in ambient air: Variation of normalized PCE over a period of 400 hours for P3HT:IC_60_BA-based devices using of TiO_2_ ETLs with and without N, H doping and N,H codoping.

Apart from the increased efficiency the high long term stability under ambient air of organic solar cells is highly desirable and was verified in the case of H doped and N,H codoped TiO_2_ embedding devices (refer to Fig. 8). The improved stability of the doped samples can be partly explained by the fact that H dopants terminate dangling bonds preventing adsorption of oxygen and moisture on the TiO_2_ surface^47^. Note that the devices were intentionally un-encapsulated so that they were exposed to ambient conditions (moisture and oxygen) throughout the aging study.

Finally, to evaluate our approach in cases where lower thermal budget is necessary, we fabricated P3HT:IC_60_BA-based OSCs using TiO_2_ ETLs post-annealed at 300 °C for 30 min to obtain H, N, or N,H codoping (refer to Fig. S9). It is observed that annealing in hydrogen containing environment induces significant enhancement in the device performance as compared to the reference cell while nitrogen annealing of TiO_2_ causes a small reduction in the device photocurrent and FF. Those results represent an indication that the intercalation of significant amount of H dopants in TiO_2_ is an effective way for boosting its electron transport capability for application in organic photovoltaics and related cells. Note that, our group has recently demonstrated the beneficial effect of H doping of ZnO (annealed at 300 °C in hydrogen environment) for organic solar cells application^32^. Based on the present results, an extensive study regarding N, H doping and N,H codoping of ZnO and influence on organic optoelectronics seems necessary and is currently carried out.

In the present study, H and N doped and codoped anatase TiO_2_ was systematically investigated using a range of experimental techniques and DFT calculations. DFT calculations reveal that the N_i_H_i_, N_i_H_O_ and N_Ti_H_i_ defects are bound, although the latter two defects require vacancies and this may hinder their formation. Conversely, H_i_ do not bind together to form extended H clusters. It is therefore expected that in codoped anatase there will be a significant concentration of N_i_H_i_ and possibly N_i_H_O_ and N_Ti_H_i_ provided that there exist vacancies so that H_O_ and N_Ti_ may form. The experimental results indicate that the formation N_i_H_i_ is more probable since they do not create deep defect states which are expected to act as hole recombination centers. H doping and N, H codoping both boost the efficiency as well as the ambient stability of organic solar cells based on P3HT:IC_60_BA. H doping and codoping with N offer several advantages including increase electron conductivity and less density of surface traps and defects at grain boundaries. The combined effects resulted in a remarkable enhancement of V_oc_, J_sc_ and FF in H doped N,H codoped TiO_2_-based OSCs.

## Methods

### Titanium Oxide Layer Preparation

FTO-coated glass (Pilkington TEC 15, <15 Ohms/sq) was cleaned by sonication in detergent solution (Hellmanex III, Hellma Analytics), water and ethanol, followed by treatment in an oxygen plasma for 5 min. A solution of 13 µL concentrated aqueous HCl in 5 mL of dry isopropanol was slowly added to a stirred solution of titanium isopropoxide (711 mg, 2.5 mmol) in 5 mL of dry isopropanol. The cleaned substrates were spin-coated at 2000 rpm with this titania precursor solution and immediately placed on a hotplate at 150 °C. Subsequently, the samples were calcinated at 500 °C for 45 min (1 h ramp). N and H doped and codoped TiO_2_ were obtained after annealing in nitrogen, hydrogen and forming gas environment at 550 °C for 1 hour. The annealing was made in a home-made furnace equipped with a quartz chamber with a graphite susceptor on which samples were placed. The susceptor was radiatively heated by three tungsten-halogen lamps of 1000 W each. The temperature of the susceptor (and therefore of the samples on it) was controlled with an automatic temperature control system, which was receiving feedback from a thermocouple positioned in a hole on the graphite and was controlling the power of the lamps. After loading samples, the chamber was evacuated down to 2 10^−2^ Torr. Then a nitrogen stream was allowed to flow through it to maintain a pressure of 0.1 Torr and the temperature was raised to the desired point (550 °C). After the preset temperature was reached, the chamber was evacuated again down to 2 10^−2^ Torr and a stream of the doping gas (hydrogen or forming gas or pure nitrogen) was introduced in it at a pressure of 1 Torr. At the end of the annealing the chamber was evacuated from the doping gas, the heating lamps were turned off and the samples were left to cool down to 70 °C under 0.1 Torr of nitrogen.

### Device Fabrication

Organic solar cells were fabricated on 40 nm thick TiO_2_ films (as-deposited or doped), which served as the electron transport layers, deposited on FTO coated glass substrates, as described above. The active layer consisted of a P3HT:IC_60_BA blend (17 mg ml^−1^ for P3HT, 17 mg ml^−1^ for IC_60_BA in 1,2-dichlorobenzene). After spin coating at 800 rpm for 30 sec the photoactive layers were left to dry for 30 min and then annealed at 150 °C for 15 min. Note that all depositions and thermal annealing treatments were carried out in the inert environment of an argon filled glove-box. Then, an approximately 20 nm-thick under-stoichiometric molybdenum oxide (MoO_3_) layer was deposited on top of the active layer, using a previously reported method, to serve as the hole transport/extraction material^48,49^. The devices were completed with a 150 nm thick aluminium anode, deposited in a dedicated thermal evaporator at a pressure of 10^–6^ Torr through a shadow-mask, which defined the device active area to be equal to 12.56 mm^2^. The devices were then measured in air at room temperature without additional encapsulation. P3HT was purchased from Rieke metals and IC_60_BA was purchased from Solenne.

### Measurements and Instrumentation

XPS and UPS were recorded by Leybold EA-11 electron analyzer operating in constant energy mode at pass energy of 100 eV and at a constant retard ratio of 4 eV for XPS and UPS, respectively. All binding energies were referred to the C 1 s peak at 284.8 eV of surface adventitious carbon. The X-ray source for all measurements was an unmonochromatized Mg Kα line at 1253.6 eV (12 keV with 20 mA anode current). The valence band spectra of TiO_2_ samples were evaluated after recording the UPS spectra of approximately 40 nm thick films deposited on an FTO substrate. For the UPS measurements, the He I (21.22 eV) excitation line was used. A negative bias of 12.22 V was applied to the samples during UPS measurements in order to separate secondary electrons originating from sample and spectrometer and to estimate the absolute work function value from the high BE cut-off region of the UPS spectra. The analyzer resolution is determined from the width of the Au Fermi edge to be 0.16 eV. Absorption measurements were taken using a Perkin Elmer Lambda 40 UV/Vis spectrophotometer. FTIR transmission spectra were obtained on a Bruker Tensor 27 spectrometer (at 4 cm^−1^ resolution, 128 scans). Film thicknesses were measured with an Ambios XP-2 profilometer. Photoluminescence measurements on TiO_2_ were carried out using a Horiba Jobin-Yvon iHR320 Spectrometer with a He–Cd laser (325 nm) as excitation source. X-ray diffraction (XRD) measurements were performed using a Siemens D500 diffractometer with Cu-Ka radiation. Current density-voltage characteristics of the fabricated solar cells were measured with a Keithley 2400 source-measure unit. Cells were illuminated with a Xe lamp and an AM 1.5 G filter to simulate solar light illumination conditions with an intensity of 100 mW/cm^2^ (1 sun), as recorded with a calibrated silicon photodiode. To accurately define the active area of all devices, we used aperture masks during the measurements with their area equal to those of the Al contacts (12.56 mm^2^). EQE measurements were carried out using an Autolab PGSTAT-30 potentiostat, with a 300 W Xe lamp in combination with an Oriel 1/8 monochromator for dispersing the light in an area of 0.5 cm^2^. A Thorlabs silicon photodiode was used for the calibration of the spectra. All measurements were performed in air.

### Computational methodology

The calculations were performed in thermally stable anatase TiO_2_ using the plane wave DFT code CASTEP^50,51^. Exchange and correlation interactions were modelled with the corrected density functional of Perdew, Burke and Ernzerhof (PBE)^52^ in the generalized gradient approximation (GGA), with ultrasoft pseudopotentials^53^. Spin polarized calculations with the inclusion of the Hubbard U contribution were used to account for the strong Coulombic interaction of the localised electrons of the 3d orbitals of Ti. The U value was set to 8.2 eV as this brings the band gap close to the experimental one in agreement to the study of Kiarii *et al* ^54^. The plane wave basis set was set to a cut-off of 480 eV, in conjunction with a 7×7 ×7 Monkhorst-Pack (MP)^55^ k-point grid and a 108-atomic site supercell. The calculations were under constant pressure conditions. The minimum energy interstitial sites and interstitial clusters were calculated by using an extensive search of all the possible combinations. We adopted and reported only the minimum energy configurations. The calculated DOS values are given in Fig. 2 with the Fermi level set at 0 eV.

## Electronic supplementary material

Supplementary Information
