# Supplementary material for: Hydrogen and nitrogen codoping of anatase TiO2 for efficiency enhancement in organic solar cells
Source: Sci Rep. 2017 Dec 19;7:17839. doi: 10.1038/s41598-017-18051-0 (PMC5736610; doi:10.1038/s41598-017-18051-0)
Supplement: Supplementary file 1 — Supplementary Information [file 41598_2017_18051_MOESM1_ESM.pdf]

Supporting Information

Hydrogen and nitrogen codoping of anatase TiO_2 for efficiency enhancement in organic solar cells

Maria Vasilopoulou,[1,*] Nikolaos Kelaidis,[2] Ermioni Polydorou,[1,3] Anastasia Soultati,[1] Dimitris Davazoglou,[1] Panagiotis Argitis,[1] Giorgos Papadimitropoulos,[1] Dimitris Tsikritzis,[4] Stella Kennou,[4] Florian Auras,[5] Dimitra G. Georgiadou,[1,6] Stavros-Richard Christopoulos[2] & Alexander Chroneos,[2,7,*]

[1]*Institute of Nanoscience and Nanotechnology (INN), National Center for Scientific Research Demokritos, 15310 Agia Paraskevi, Athens, Greece*

[2]*Faculty of Engineering, Environment and Computing, Coventry University, Priory Street, Coventry CV1 5FB, United Kingdom*

[3]*Department of Physics, University of Patras, 26504, Patras, Greece*

[4]*Department of Chemical Engineering, University of Patras, 26504, Patras, Greece*

[5]*Cavendish Laboratory, University of Cambridge, Cambridge CB3 0HE, United Kingdom*

[6]*Department of Physics & Centre for Plastic Electronics, Imperial College, London SW7 2AZ, United Kingdom*

[7]*Department of Materials, Imperial College, London SW7 2AZ, United Kingdom*

*m.vasilopoulou@inn.demokritos.gr, alexander.chroneos@imperial.ac.uk

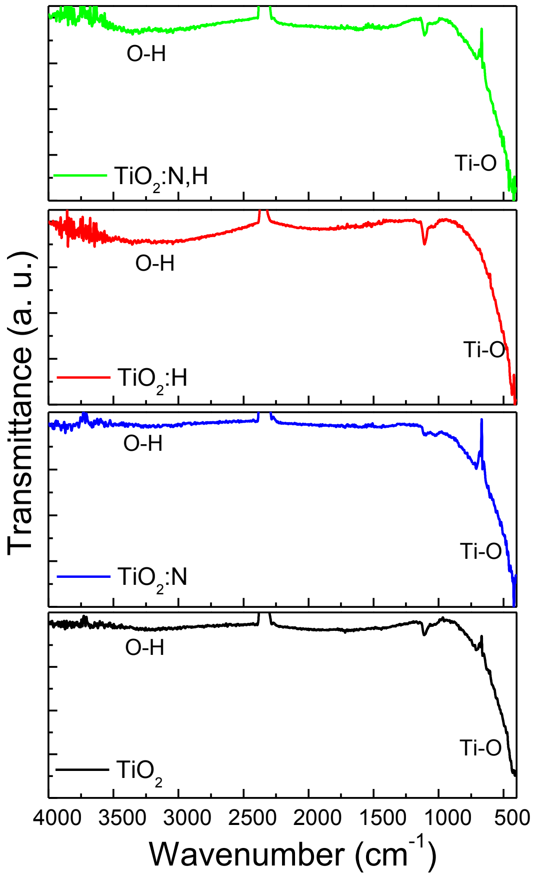

Figure S1. FTIR transmittance spectra of TiO_2 samples either as-deposited (on silicon substrates) or annealed in nitrogen, hydrogen and forming gas (containing 90% nitrogen and 10% hydrogen) at 550 ^{o}C for 1 h. The thickness of the samples was ~40 nm.

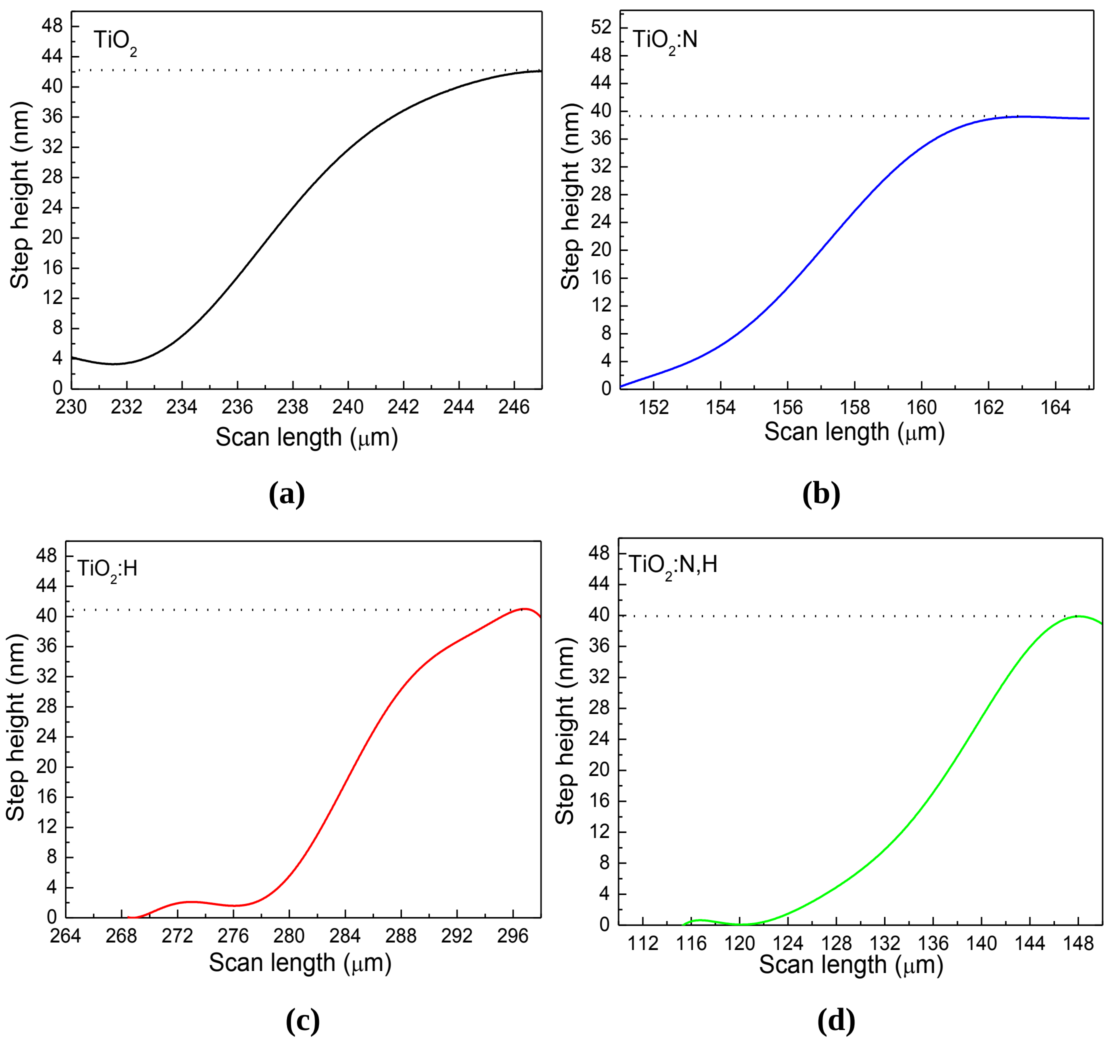

Figure S2. Profilometer thickness profile measurements of TiO_2 samples.

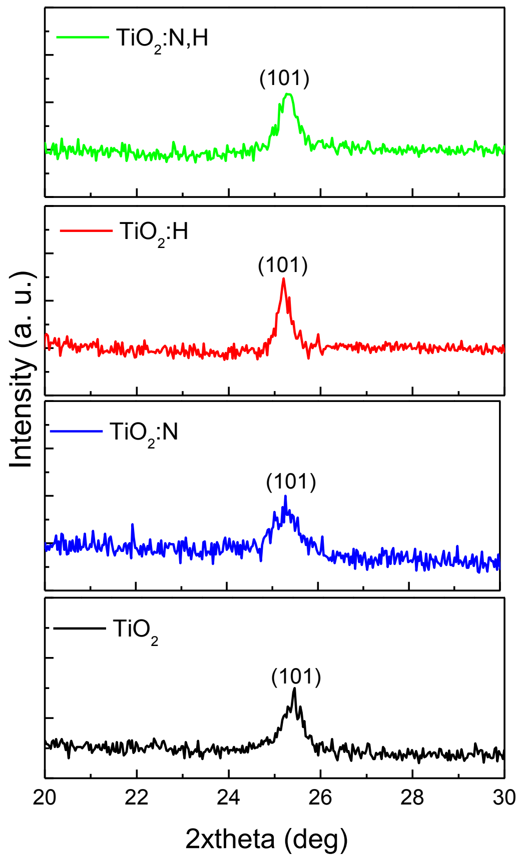

Figure S3. X-ray diffraction patterns of TiO_2 samples with a thickness of ~40 nm. The samples were either as-deposited or annealed in nitrogen, hydrogen and forming gas at 550 ^oC for 1 h.

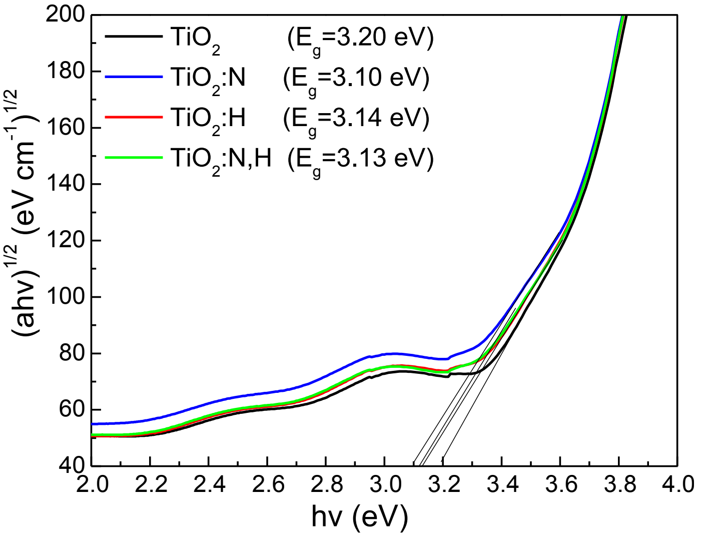

Figure S4. Tauc plots, as derived from absorption measurements, for the estimation of bandgap for the different TiO_2 samples.

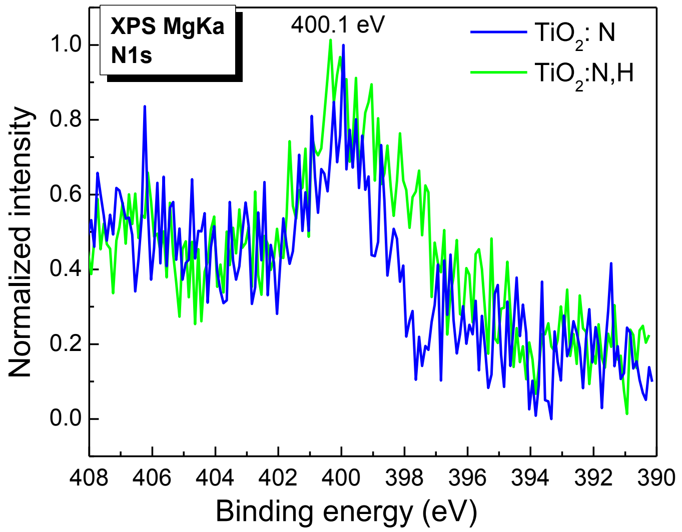

Figure S5. XPS N 1s peaks of TiO_2 samples annealed in nitrogen and forming gas environments at 550 ^{o}C for 1 hour.

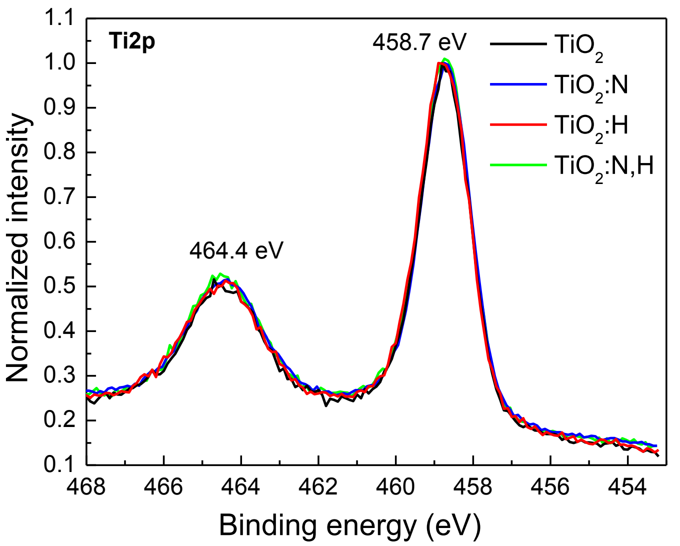

Figure S6. XPS Ti 2p peaks of TiO_2 samples annealed in different environments.

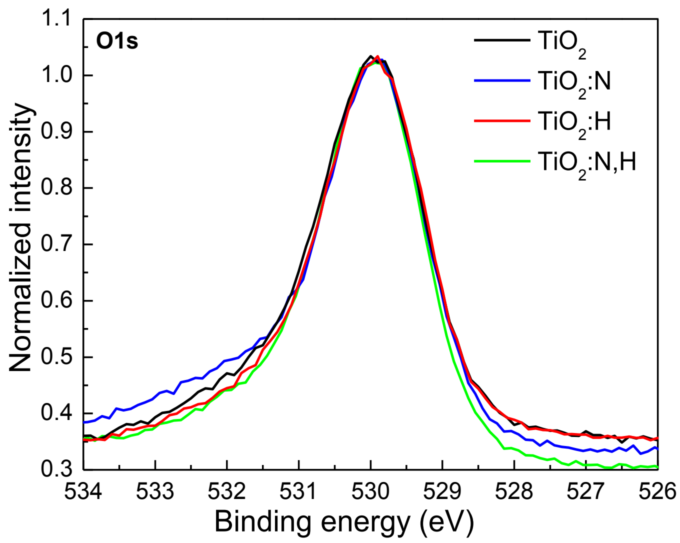

Figure S7. XPS O 1s peaks of TiO_2 samples annealed in different environments.

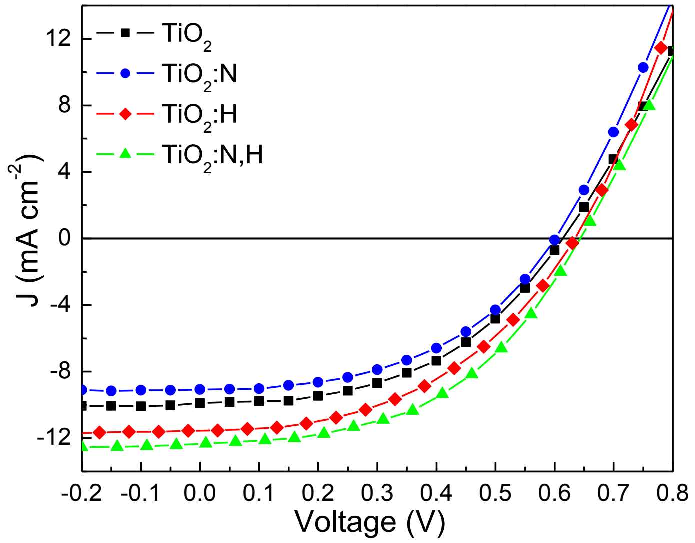

Figure S8. Current density versus applied voltage (J-V) curves taken under 1.5 AM illumination of P3HT:PC_{70}BM-based organic solar cells using N and H doped and codoped TiO_2 ETLs. The annealing in different environments was performed at 550 ^{o}C for 1 hour.

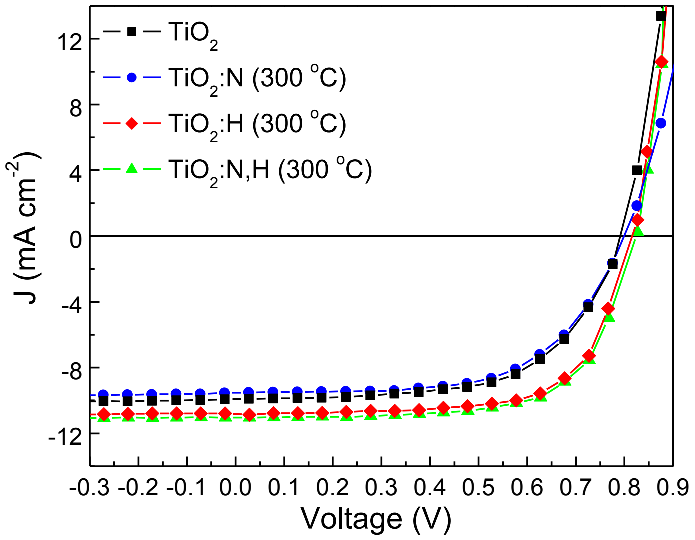

Figure S9. Current density versus applied voltage (J-V) curves taken under 1.5 AM illumination of P3HT:IC_{60}BM-based organic solar cells using TiO_2 ETLs. The TiO_2 layers were either as-deposited or annealed in nitrogen, hydrogen and forming gas at 300 ^{o}C for 30 min.
